# The Stealth Episome: Suppression of Gene Expression on the Excised Genomic Island PPHGI-1 from *Pseudomonas syringae* pv. *phaseolicola*

**DOI:** 10.1371/journal.ppat.1002010

**Published:** 2011-03-31

**Authors:** Scott A. C. Godfrey, Helen C. Lovell, John W. Mansfield, David S. Corry, Robert W. Jackson, Dawn L. Arnold

**Affiliations:** 1 Faculty of Health and Life Sciences, University of the West of England, Bristol, United Kingdom; 2 Department of Life Sciences, Imperial College London, London, United Kingdom; 3 School of Biological Sciences, University of Reading, Reading, United Kingdom; University of Toronto, Canada

## Abstract

*Pseudomonas syringae* pv. *phaseolicola* is the causative agent of halo blight in the common bean, *Phaseolus vulgaris*. *P. syringae* pv. *phaseolicola* race 4 strain 1302A contains the avirulence gene *avrPphB* (syn. *hopAR1*), which resides on PPHGI-1, a 106 kb genomic island. Loss of PPHGI-1 from *P. syringae* pv. *phaseolicola* 1302A following exposure to the hypersensitive resistance response (HR) leads to the evolution of strains with altered virulence. Here we have used fluorescent protein reporter systems to gain insight into the mobility of PPHGI-1. Confocal imaging of dual-labelled *P. syringae* pv. *phaseolicola* 1302A strain, F532 (dsRFP in chromosome and eGFP in PPHGI-1), revealed loss of PPHGI-1::eGFP encoded fluorescence during plant infection and when grown *in vitro* on extracted leaf apoplastic fluids. Fluorescence-activated cell sorting (FACS) of fluorescent and non-fluorescent PPHGI-1::eGFP F532 populations showed that cells lost fluorescence not only when the GI was deleted, but also when it had excised and was present as a circular episome. In addition to reduced expression of eGFP, quantitative PCR on sub-populations separated by FACS showed that transcription of other genes on PPHGI-1 (*avrPphB* and *xerC*) was also greatly reduced in F532 cells harbouring the excised PPHGI-1::eGFP episome. Our results show how virulence determinants located on mobile pathogenicity islands may be hidden from detection by host surveillance systems through the suppression of gene expression in the episomal state.

## Author Summary

Bacterial pathogens evolve rapidly through the transfer of large segments, or genomic islands (GIs), of DNA. We study the mobility of an island named PPHGI-1 in *Pseudomonas syringae* pv. *phaseolicola* that causes halo-blight disease of bean. The exposure of *P. syringae* pv. *phaseolicola* to plant defenses triggers the excision of PPHGI-1, creation of a circular episomal form and finally deletion of the GI or its transfer to other bacteria. We planned to examine deletion of PPHGI-1 within infected leaves, and we generated strains that expressed differently coloured fluorescent proteins from genes in the island or elsewhere on the chromosome. Loss of the specific fluorescence derived from the GI was expected to show deletion of PPHGI-1. However, collecting fluorescent and non-fluorescent bacteria showed that PPHGI-1 was usually not lost, but expressed its component genes very poorly when in the circularized state. Bacteria were therefore able to carry a hidden suite of genes that become activated when re-inserted into the chromosome. The “stealthy” movement of the island is beneficial to *P. syringae* pv. *phaseolicola* because genes on PPHGI-1 encode proteins that activate plant defenses. Similar gene silencing on episomes may occur in other pathogens and contribute to the evolution of microbial pathogenicity to animals and plants.

## Introduction

The exchange of genomic pathogenicity islands by horizontal gene transfer is recognised as a key factor in the development of new more virulent bacterial pathogens of both animals and plants [1], [2], [3], [4]. *Pseudomonas syringae* is a bacterial species sub-divided into pathovars based on host range. Infection of a wide variety of plants results in necrotic symptoms in leaves, stems, and fruit [5]. *P. syringae* pv. *phaseolicola* causes halo-blight of common bean and has emerged as a model bacterial pathogen for the analysis of the evolution of pathogenicity [6], [7], [8], [9].

The primary mechanism of plant resistance against *P. syringae* is a basal defense response that is induced upon detection of conserved microbe associated molecular patterns (MAMPS) [10]. Pathovars of *P. syringae* are able to overcome MAMP triggered defense by delivering into the plant cytoplasm an array of effector proteins that inactivate surveillance mechanisms and signal transduction pathways, thereby allowing bacterial multiplication [11], [12], [13]. Many plants have evolved resistance proteins that recognise a subset of these effector proteins, termed avirulence (Avr) proteins, which trigger a hypersensitive resistance response (HR) leading to programmed cell death at infection sites and the restriction of colonisation [14], [10].

Molecular analysis of genetic interactions between *P. syringae* pv. *phaseolicola* and its host bean has led to the identification and characterization of a number of bacterial *avr* genes and plant *R* genes [7]. One of these *avr* genes, *avrPphB* (syn. *hopAR1*), encodes an effector protein that induces an HR in bean cultivar (cv.) Tendergreen (TG) which carries the *R3* resistance gene. In *P. syringae* pv. *phaseolicola* strain 1302A, *avrPphB* is located on a 106 kb genomic island (GI) designated PPHGI-1 [6]. The similarity of PPHGI-1 to other integrative and conjugative elements (ICElands) suggests that the island integrates and excises at *att* sites within the tRNA locus found at its borders, via an episomal circular intermediate [15], [16]. PPHGI-1 can be acquired by transformation between strains of *P. syringae* pv. *phaseolicola* [17] and is physically lost from cells of *P. syringae* pv. *phaseolicola* during infection of TG undergoing the R3*-*AvrPphB mediated HR [18], [6]. Following excision from the chromosome, a critical step in the mobilization of PPHGI-1 is formation of the circular episome that is capable of limited replication [17], [19].

The plant apoplast comprises the intercellular space surrounding plant cells where metabolic and physiological processes relating to cell wall biosynthesis, nutrient transport, and stress responses occur [20]. It is also the site of colonisation for *P. syringae* pv. *phaseolicola* which obtains nutrients directly from apoplastic fluid for *in planta* survival and multiplication. We have previously shown that complete PPHGI-1 transfer can occur between *P. syringae* pv. *phaseolicola* 1302A and *P. syringae* pv. *phaseolicola* 1448A, both in the leaf apoplast and in extracted *P. vulgaris* TG and Canadian Wonder (CW) apoplastic fluids [17]. We hypothesized that such a transfer requires four distinct processes: excision of the island from the chromosome, release of the circular episome from the bacterium, relocation into competent bacterial cells and finally integration into a specific *att* site within the genome.

As GI movement is responsible for major evolution in *P. syringae* pv. *phaseolicola* [21], [8], understanding the stages involved in GI transfer between bacterial strains will greatly aid our understanding of the evolutionary process. Although we know that exposure to the plant's immune response activates excision and transfer of the PPHGI-1, there is no information available on the dynamics of island loss from colonies of bacteria within infected tissues. In order to address the spatial dynamics of excision in the context of the emergence of new strains of *P. syringae* pv. *phaseolicola* at microsites within infected tissues, we developed fluorescent protein-based systems to monitor the movement of PPHGI-1 in and out of the genome. In addition, using a combination of fluorescence-activated cell sorting and quantitative PCR, we found that gene expression from the excised PPHGI-1 episome was reduced so that absence of fluorescence did not reflect loss of the GI, but its excision and circularization. Switching off gene expression after excision of pathogenicity islands may be a widespread phenomenon allowing bacterial products to be hidden from host defenses and thereby facilitating “stealthy” transfer of genes encoding virulence factors that may be of benefit under new infection conditions.

## Results and Discussion

### Creation of dual-labelled fluorescent *P. syringae* pv. *phaseolicola* strains

**Figure 1:**
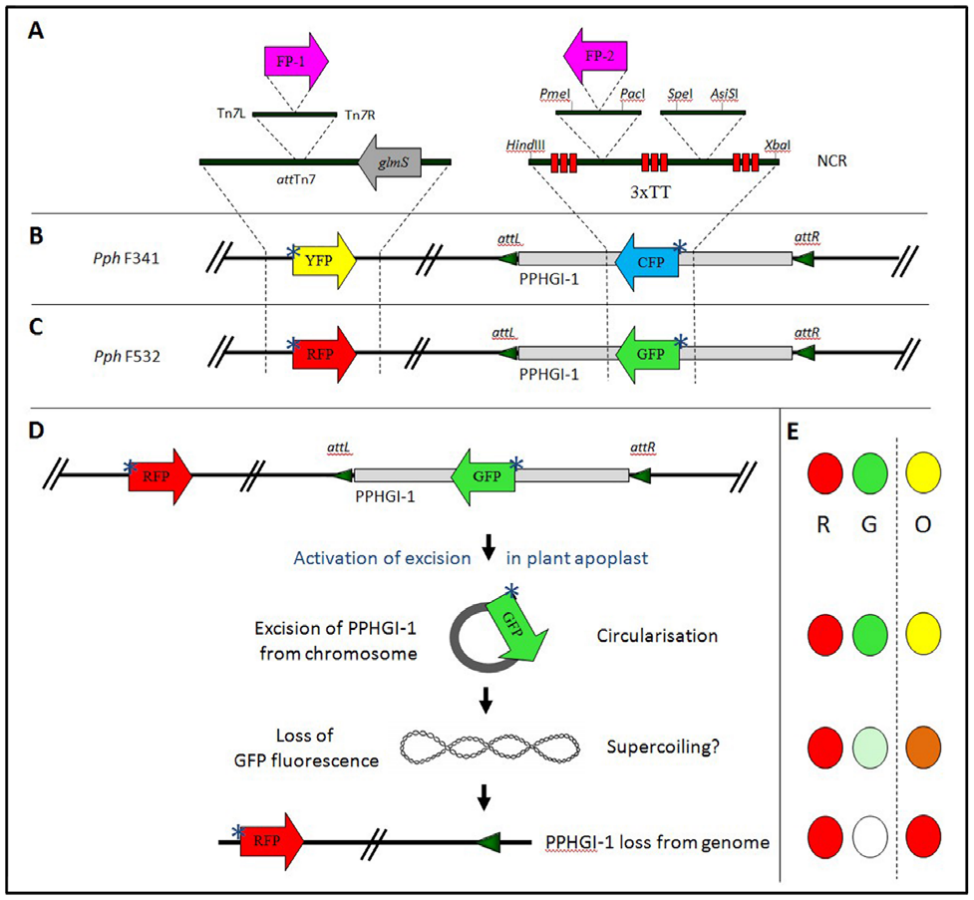
Generation of strains of *P. syringae* pv. *phaseolicola* 1302A tagged with fluorescent proteins. **A**. Introduction of constitutive fluorescent reporter genes separately into the *P. syringae* pv. *phaseolicola* 1302A chromosome and PPHGI-1 genomic island to create **B**. *P. syringae* pv. *phaseolicola* F341 containing eYFP introduced into the chromosome upstream of *glmS* using a Tn*7* delivery system and eCFP within the non-coding region of PPHGI-1 and **C**. *P. syringae* pv. *phaseolicola* F532 with dsRFP in the chromosome and eGFP in PPHGI-1. **D**. Schematic depiction of PPHGI-1 excision (with loss of GFP expression) from *P. syringae* pv. *phaseolicola* F532 within the plant environment with **E**. fluorescence detectable in channels R, dsRFP; G, eGFP and O, with overlapping channels. Loss of eGFP fuorescence would also report the deletion of PPHGI-1 from bacteria. Abbreviations are as follows: FP-1, fluorescent protein either eYFP or dsRFP; FP-2, fluorescent protein either eGFP or eCFP; TT, transcriptional terminators; NCR, non-coding region in PPHGI-1; ‘*’ denotes constitutive reporter expression. *Spe*I - *Asi*SI region is a secondary rare-cloning site. The position on PPHGI-1 of genes used in this study are avrPphB 17,471–18,274 bp and xerC 105,384–106,805 bp [6]. The genes for the fluorescent proteins have been inserted in non-coding region at position 55,660–56, 299 bp.

We aimed to visualise PPHGI-1 dynamics in the plant and *in vitro* using constitutively expressed fluorescent markers. Strains of *P. syringae* pv. *phaseolicola* 1302A were generated expressing fluorescent proteins encoded from PPHGI-1 (e.g. PPHGI-1::eGFP) and also from other regions of the chromosome as illustrated in Figure 1A. *P. syringae* pv. *phaseolicola* F341 contained chromosomal eYFP and PPHGI-1::eCFP (Figure 1B) and *P. syringae* pv. *phaseolicola* F532 chromosomal dsRFP and PPHGI-1::eGFP (Figure 1C). Loss of PPHGI-1 was expected to lead to loss of the GI encoded fluorescence but retention of emission from the other fluorophore (Figure 1D, E). *P. syringae* pv. *phaseolicola* F341 and *P. syringae* pv. *phaseolicola* F532 also displayed wildtype *P. syringae* pv. *phaseolicola* 1302A phenotypes; for example *in vitro* and *in planta* growth rates, plasmid profiles, and equal rates of PPHGI-1 loss when passaged through TG (data not shown).

### Confocal microscopy of dual-labelled fluorescent *P. syringae* pv. *phaseolicola* strains in leaves

**Figure 2:**
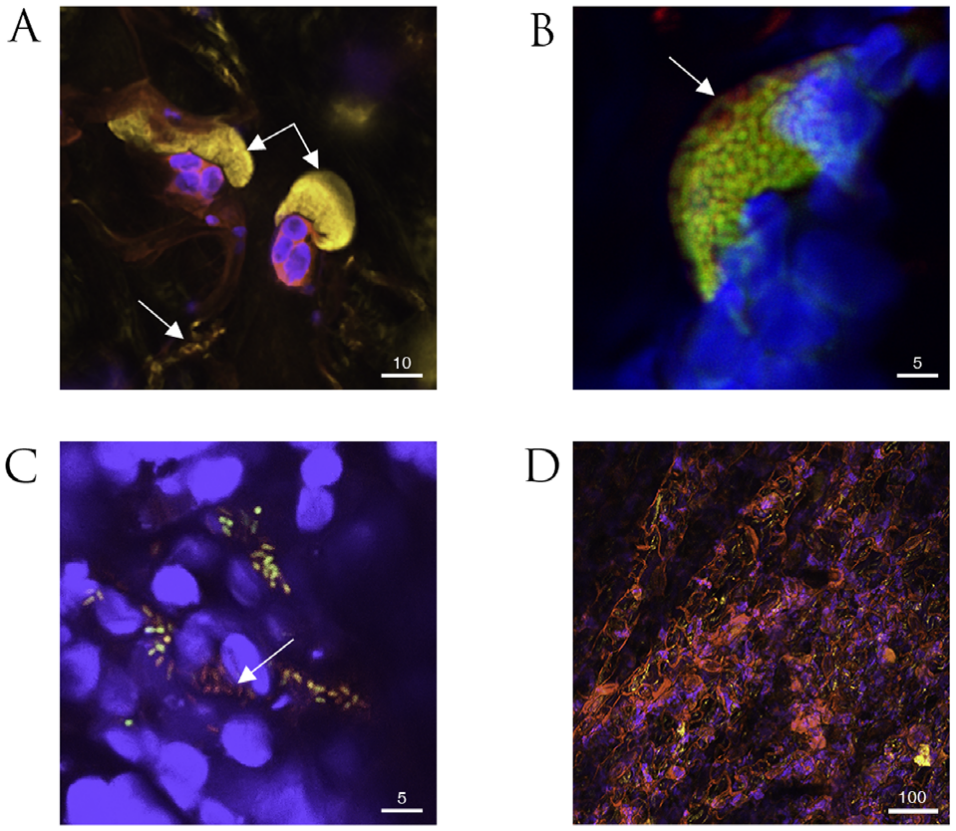
Confocal microscopy of fluorescently labeled *P. syringae* pv. *phaseolicola* 1302A strains in bean leaf tissue. **A**. Colonies of *P. syringae* pv. *phaseolicola* F341 expressing eYFP attached to mesophyll cells undergoing the HR (double arrow) and also dispersed individual bacteria (single arrow) in cv. Tendergreen (TG). **B**. Colony of *P. syringae* pv. *phaseolicola* F532 containing chromosomally expressed dsRFP and eGFP in the mobile GI, PPHGI-1, exhibit peripheral loss of eGFP fluorescence (arrow) in TG. **C**. Dispersed cells from *P. syringae* pv. *phaseolicola* F532 colonies in cv. Canadian Wonder (non-HR) include individuals without eGFP, but expressing chromosomally encoded dsRFP (arrowed). **D**. Severe R3-AvrPphB mediated hypersensitive response (HR) in TG in the presence of *P. syringae* pv. *phaseolicola* F341 (high inoculum, 8×10^7^ CFU/ml) causes the release of numerous autofluorescent compounds (pink coloration). The autofluorescence of the HR compromises visualization of introduced fluorophores. All images were assigned false colours to represent selective emission detection (pink, auto fluorescence emission from collapsed plant tissue; blue, plant mesophyll cell chloroplast fluorescence; yellow, eYFP; green/yellow, overlay of co-expressing *P. syringae* pv. *phaseolicola* F532 dsRFP and eGFP; and red, dsRFP emission only). All images were taken 72 h after inoculation. Size bars are given in µm.

We have previously described the use of bacteria separately labeled with fluorophores for confocal microscopy of infected leaves [22]. With the strains constructed to express two fluorophores, we planned to follow loss of PPHGI-1 occurring at microsites within challenged leaves and in particular during the HR in TG. Colonies of *P. syringae* pv. *phaseolicola* develop in the intercellular spaces, typically attached to leaf mesophyll cells. A feature of tissue undergoing the HR in TG was the dispersal of colonies to reveal individual bacterial cells as illustrated in Fig. 2A. When *P. syringae* pv. *phaseolicola* F532 was infiltrated into TG or CW leaves, colonies frequently showed peripheral loss of PPHGI-1::eGFP fluorescence (Figure 2B). Dispersed bacterial cells of F532 in TG and around the cut edges of leaf samples in both cultivars also displayed mixed fluorescence (Fig. 2C). The loss of fluorescence from colonies was more easily detected in the susceptible variety CW, rather than resistant TG, because imaging whole colonies after the HR was partly compromised by the accumulation of autofluorescent plant-derived metabolites (Figure 2D, [22]). Limiting initial *P. syringae* pv. *phaseolicola* inoculum concentrations in TG was effective in minimizing the spread of the HR and was necessary to avoid spectral overlap of dsRFP and HR autofluorescence.

Our observations with F532 highlighted that the outer layer(s) of bacteria within established colonies (in both TG and CW leaves) preferentially exhibit loss of PPHGI-1::eGFP fluorescence. Such colony differentiation may be due to the activities of diffusion mediated gradients similar to those proposed in biofilm models [23], where the colony periphery receives higher concentrations of diffused substrates (e.g. nutrients, oxygen, environmental stimuli, and/or antimicrobial compounds) and therefore exhibits differing metabolic activity compared with the colony centre [24]. Higher rates of *in planta* loss of PPHGI-1::eGFP fluorescence from the periphery of F532 colonies may be due to higher levels of environmental cue(s) from the *P. vulgaris* apoplast and/or due to a higher metabolic activity due to enhanced nutrient acquisition. Significantly, loss of PPHGI-1 derived fluorescence appeared to be as frequent in the susceptible CW leaves as in the resistant TG which underwent the HR. By contrast, previous work had shown that cells that have lost PPHGI-1 are more readily selected during the HR than during the development of infection in susceptible tissues such as CW leaves [6], [19].

### Loss of eGFP fluorescence from F532 is observed in apoplastic fluids

**Figure 3:**
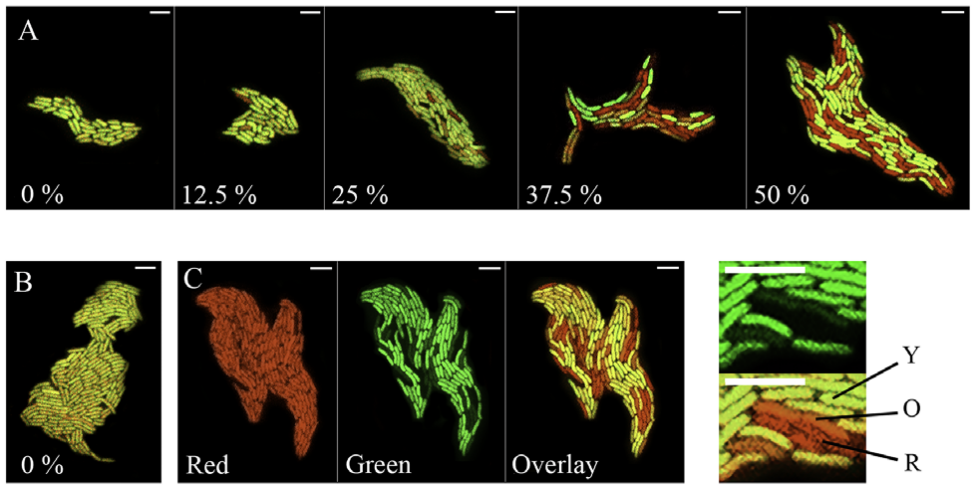
Confocal monitoring of fluorescence from PPHGI-1::eGFP in *P. syringae* pv. *phaseolicola in vitro.* * P. syringae* pv. *phaseolicola* F532 was grown for 48 h on extracted Tendergreen apoplastic fluid (TG-apo) in agarose (1.4%). **A**. After 48 h, eGFP loss occurs from *P. syringae* pv. *phaseolicola* F532 only in high concentrations of TG-apo mixed with M9 minimal medium, % relates to the amount of apoplastic fluid in the medium. **B**. *P. syringae* pv. *phaseolicola* F532 shows no loss of PPHGI-1::eGFP when grown on 100% M9 (0% apo) for 144 h. **C**. Illustrates how images were produced by recording the: red channel; green channel; and then overlaying the two channels. Close up images with lines denote cells with varying eGFP emission quantum yields compared with cells expressing only eGFP in the upper panel (Y, denotes yellow cells with equal emission levels of eGFP and dsRFP; O, denotes orange cells with reduced eGFP emission; and R, denotes red cells which have no detectable eGFP emission). Note that all cells present expressed dsRFP at equal intensity as in Ci. All images were assigned false colour (red, dsRFP; green, eGFP; yellow, overlay of dsRFP and eGFP*)*; size bars, 5 µm.

Lovell *et al*. [17] demonstrated that apoplastic fluids extracted from leaves provide a medium that promotes the mobility of PPHGI-1. To determine if PPHGI-1::eGFP fluorescence was lost from F532 *in vitro*, the strain was grown on apoplastic fluids extracted from leaves of CW and TG, mixed with agarose and coated on microscope slides to facilitate microscopy. Fluorescence was lost rapidly from colonies grown on apoplastic fluids but not from M9-based media (Figure 3). Increasing the concentration of either CW or TG apoplastic fluid in the agarose slide medium caused a corresponding increase in the frequency of loss of fluorescence due to PPHGI-1::eGFP (Figure 3A). To confirm that loss of PPHGI-1 encoded fluorescence was not specific to strain F532, *P. syringae* pv. *phaseolicola* F341 was examined in the same way and rates of loss of PPHGI-1::eCFP were comparable to those found for PPHGI::eGFP in F532 (data not shown). Clearly, some component(s) of apoplastic fluid from either cultivar promoted loss of fluorescence of respectively labeled PPHGI-1 constructs, suggesting that the occurrence of the HR is not required for the release of inducing metabolites from plant cells.

### Loss of PPHGI-1::eGFP fluorescence is not always due to loss of the GI from the bacterial cell

To determine whether or not changes in PPHGI-1::eGFP fluorescence were due to cellular loss of PPHGI::eGFP, F532 cells were first grown on M9/TG agarose slides. Following confocal imaging to confirm loss of PPHGI-1::eGFP fluorescence from some cells but positive dsRFP fluorescence, bacteria from the imaged agarose slide were resuspended in ¼ Ringers and cultured to individual colonies on KB agar. All resulting colonies were found to express eGFP. Furthermore, 200 colonies were analysed for the presence of *avrPphB* by the TG pod-stab pathogenicity assay and all colonies were found to cause the HR. This suggested that, despite the temporary loss of expression of the PPHGI-1::eGFP construct after growth on apoplastic fluids, PPHGI-1 was retained and functional.

### FACS separation of *P. syringae* pv. *phaseolicola* F532 populations based on eGFP fluorescence

**Figure 4:**
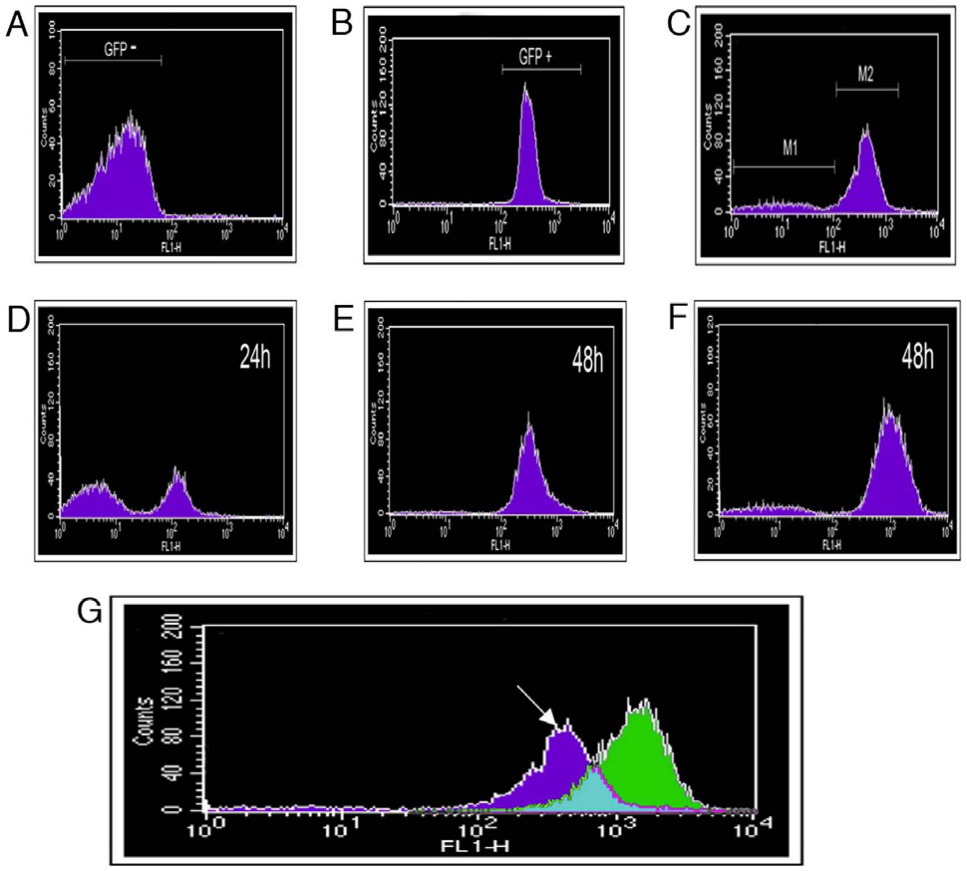
Fluorescence-activated cell sorting (FACS) separation of *P. syringae* pv. *phaseolicola* cells based on fluorescent protein expression. Histograms record fluorescence detected by the FL1-H detector after analysis of 200,000 particles (events). Analysis of **A**. *P. syringae* pv. *phaseolicola* 1302A grown in M9, 48 h. **B**. *P. syringae* pv. *phaseolicola* SG248 with constitutive eGFP introduced chromosomally, grown in M9, 48 h. and **C**. *P. syringae* pv. *phaseolicola* F532 grown in M9/TG apoplastic fluid (1:1) for 48 h. Gating was performed to collect equal populations of (M1) *P. syringae* pv. *phaseolicola* F532 that did not express eGFP and (M2) eGFP expressing *P. syringae* pv. *phaseolicola* F532. Gated subpopulations were then grown in LB to investigate changes in PPHGI-1::eGFP expression. **D**. Population M1, after incubation for 24 h, note the presence of eGFP fluorescing bacteria, **E**. M1 after 48 h and **F**. Population M2 after 48 h. **G**. An overlay of GFP detection from F532 grown in LB (Green) and F532 grown in M9/TG apoplastic fluid (1:1) for 48 h (dark blue). Counts refer to fluorescence intensity. The arrow denotes region corresponding to F532 cells with reduced PPHGI-1::eGFP expression. Similar background fluorescence was observed in all media.

To investigate the apparent silencing of eGFP, fluorescence-activated cell sorting (FACS) was employed to obtain pure populations of F532 with or without eGFP fluorescence; F532/GFP+ and F532/GFP-, respectively (Figure 4). We first tested the suitability of FACs to differentiate the fluorescent Pseudomonads. Low background was recorded in 1302A without added fluorophores (Fig. 4A) but this did not compromise the strong signal from eGFP (Fig. 4B).

To collect eGFP fluorescing and non-fluorescent bacteria, F532 cultures were grown (48 h) in stationary M9/TG apoplastic fluid (1∶1) broths and 200,000 particles (events) were collected for both F532/GFP+ and F532/GFP- cells. Sub-populations were serially diluted and grown on KB+Gm and the viable cells recovered were: F532/GFP+ (2.64×10^4^ CFU/ml) and F532/GFP- (1.06×10^4^ CFU/ml). The decrease in viable CFU/ml from F532/GFP- events compared to F532/GFP+ was likely due to detection of non-fluorescent particles, from either non-viable F532 cells and/or plant debris from apoplastic fluid.

The separated sub-populations of F532/GFP- and F532/GFP+ cells were monitored for further changes in PPHGI-1::eGFP fluorescence over a 48 h period (in LB, static at 25°C, Figs. 4C–F). If PPHGI-1::eGFP excision and re-insertion into the chromosome was a dynamic process, we expected to see a reversion of the F532/GFP- (Figure 4 C, M1) sample to form a major population of F532/GFP+ cells, with a minor population of F532/GFP- cells. As predicted, F532/GFP- cells were observed to revert to a population of predominantly F532/GFP+ cells (Figure 4D, E) over a 48 h period. Also, F532/GFP+ cells from gated population M2 (Figure 4C) formed a sub-population of F532/GFP- cells (M1-like, Figure 4F) indicating the continued mobility of PPHGI-1::eGFP in F532. It is interesting that after 48 h both FACS sub-populations reverted back to the same general population structure (as in Figures 4E and 4F) suggesting that there may be an equilibrium between F532 cells containing PPHGI-1 in the chromosome (expressing eGFP) and F532 cells containing the excised PPHGI-1 (not expressing eGFP).

Figure 4G illustrates the changes in patterns of sorted fluorescent particles comparing F532 grown in apoplastic fluid which produces a broader GFP detection spectrum than F532 grown in LB broth. This result correlates with different levels of PPHGI-1::eGFP fluorescence (whilst dsRFP remained constant) observed in F532 cells examined by confocal microscopy (e.g. Figure 3C).

### qPCR reveals correlation between increased production of the circular episome of PPHGI-1 and decreased gene expression in F532/GFP- cells

**Figure 5:**
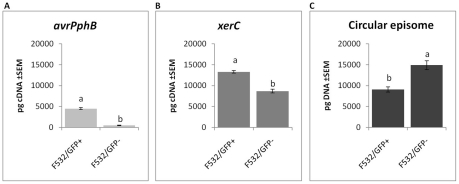
Gene expression and circular episome detection in sub-populations of *P. syringae* pv. *phaseolicola*. Analysis of FACS-separated sub-populations of F532 expressing GFP (F532/GFP+) and F532 without GFP fluorescence (F532/GFP-) shows: **A**. F532/GFP- has effectively no expression of *avrPphB*, **B**. *xerC* expression is also reduced in F532/GFP-; and **C**. PPHGI-1 circular episome formation is significantly greater in F532/GFP- cells than F532/GFP+ cells. All data were standardized by simultaneous qPCR analysis of *gyrB* expression and error bars represent standard error of the mean of three experimental replicates. Letters above bars indicate significant differences at p<0.05 assessed with Student's t test.

We had shown that loss of PPHGI-1::eGFP fluorescence was often not due to the deletion of PPHGI-1 from F532. Therefore, we investigated whether down-regulation of gene expression occurs after GI excision and circularization, thereby accounting for loss of PPHGI-1::eGFP fluorescence. qPCR was performed on FACS separated F532 populations to determine differences between F532/GFP- and F532/GFP+ cells by comparison of: a) the amount of PPHGI-1 forming a circular PPHGI-1 episome (*CE*); and b) the expression levels of two genes contained within PPHGI-1 (*avrPphB* and *xerC*). Results reveal that there is increased formation of *CE* in F532/GFP- cells, but greatly reduced expression of *avrPphB* and *xerC* (Figure 5). We conclude that loss of fluorescence from F532 does not, therefore, usually indicate loss of PPHGI-1, but is in fact reporting creation of the excised *CE* from which gene expression is greatly reduced. Such reduced expression of GI genes could be explained by transcriptional and translational down-regulation due to supercoiling of DNA (Figures 1D & E) [25], [26] and/or possible association of histone-like proteins influencing gene expression [27], [28].

### Frequency of occurrence of the circular episome

**Figure 6:**
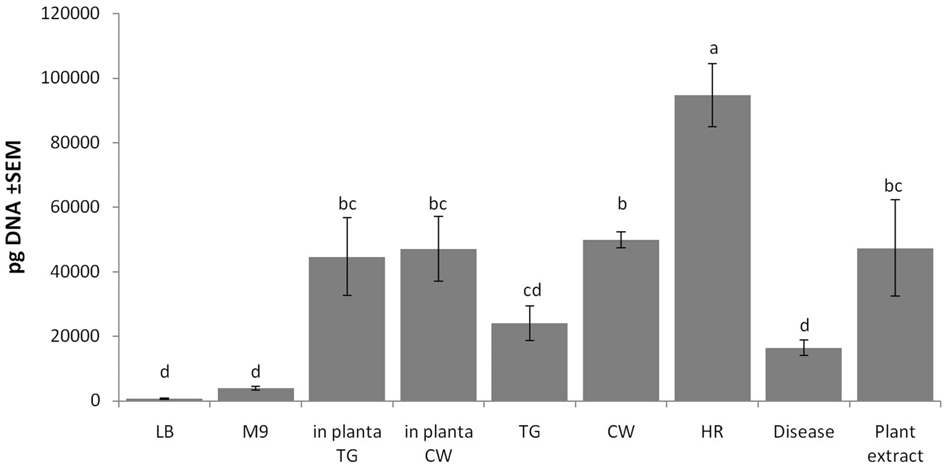
Environmental effects on the detection of the PPHGI-1 circular episome determined by qPCR. Abbreviations are as follows: LB, Luria Bertani medium; M9, minimal medium; TG, apoplastic fluid extracted from *P. vulgaris* cv. Tendergreen (TG); CW, apoplastic fluid extracted from *P. vulgaris* cv. Canadian Wonder (CW); HR, apoplastic fluid extracted from TG leaf undergoing *R3*-AvrPphB mediated HR; Disease, apoplastic fluid extracted from CW leaf undergoing *P. syringae* pv. *phaseolicola* 1302A induced disease; Plant extract, homogenised TG leaf in M9; *in planta,* within either *P. vulgaris* TG or CW leaf after infiltration. qPCR was performed after 5 h incubation *in vitro* or after plant inoculation. All data were standardized by simultaneous qPCR analysis of *gyrB* expression and error bars represent standard error of the mean of three experimental replicates. Letters above bars indicate significant differences at p<0.05 assessed with Student's t test.

It is well established that PPHGI-1 is lost from 1302A cells during the HR in bean leaves. The antimicrobial conditions generated by the HR appear to select for bacteria lacking the avirulence gene *avrPphB* [6], [21], [19]. The first stage in deletion of PPHGI-1 is its excision from the chromosome and the formation of the *CE* that was first detected by PCR in bacteria grown in LB [6], [21], [19]. Here we applied the more stringent method of qPCR to confirm the presence of *CE* in F532/GFP- cells (Fig. 5). The high frequency of occurrence of bacteria down-regulating gene expression on PPHGI-1 detected *in vitro* in apoplastic fluids suggested that bacteria harbouring the excised episome might be far more common in certain environments. We therefore used qPCR to examine the levels of PPHGI-1 *CE* in bacteria grown under various conditions for 5 h (Fig. 6).

The experiment revealed strikingly higher levels of *CE* in bacteria inoculated into bean leaves than grown in LB or minimal media. At the 5 h time point, however, there was no difference in *CE* concentration in resistant TG, or susceptible CW leaves. As expected, *CE* was present at high levels in bacteria incubated in apoplastic fluids. The highest amounts were found after growth in fluids recovered from leaves undergoing the HR and the lowest from susceptible, diseased tissue. The addition of whole leaf extracts to M9 also created conditions favouring formation of the *CE*.

Taken together these results suggest that excision of PPHGI-1 is promoted by a plant factor(s) but there is little differentiation between susceptible and resistant plant tissues before infection. The occurrence of the HR does appear to enhance excision and formation of the circular intermediate, but not to such an extent that would explain the high frequency of loss of PPHGI-1 during the HR [6]. We conclude that *P. syringae* pv. *phaseolicola* PPHGI-1 excision, circularisation and re-insertion is a very dynamic process necessary to maintain PPHGI-1 replication within *P. syringae* pv. *phaseolicola* populations. This would explain the variance in detectable levels of PPHGI-1::eGFP fluorescence from adjacent cells during confocal imaging (e.g. Figure 3C) where images obtained are most likely ‘snapshots’ of F532 cells at different stages of PPHGI-1::eGFP excision. It is highly likely that the excision of PPHGI-1 may interfere with the timeline of eGFP transcription, translation, protein folding, maturation and degradation – all ultimately affecting the levels of PPHGI-1::eGFP fluorescence.

The reduced expression of genes on the *CE*, notably *avrPphB*, would provide a selective advantage for the bacterial cells allowing their increased multiplication in resistant TG tissues and favouring subsequent selection of bacteria that had lost PPHGI-1. Alternatively, the multiplication of bacteria maintaining the silenced PPHGI-1 may prove advantageous should they be spread to other plants lacking the *R3* resistance gene. The re-integration of PPHGI-1 into the chromosome would lead to a return to normal levels of expression of genes encoded on the GI.

In our previous work on the mobility of PPHGI-1 we used failure to induce the HR as an indication of loss of the island and confirmed this result by PCR-based diagnostics [6]. Our results on the suppression of gene expression from the circularised episome show that there is a complex pattern of selection occurring within the infected plant. Because we have demonstrated movement of the island both out of and back into the chromosome, there must be a resultant fluctuation in levels of expression of *avrPphB*. It would only be those bacteria that have physically lost PPHGI-1 that would retain virulence during colonisation and presumably break out from sites undergoing the effector-triggered HR. In order to dissect the dynamics further we need to investigate the mechanisms controlling deletion of the island in more detail.

The initial aim of our work was to use confocal microscopy to examine PPHGI-1 deletion at micro-sites within infected leaves. We were only partially successful because of the suppression of gene expression from the excised episome. However, our findings may be of more general significance to our understanding of microbial pathogenicity. Dynamic excision and reinsertion of GIs has been described in other *Pseudomonas* species [29], [30]. We suggest that the silencing of genes carried on GIs following excision from their chromosomal location may be an important strategy utilized not only by *P. syringae* pv. *phaseolicola* but also by other bacterial pathogens of plants and animals. Switching off gene expression after GI excision is a novel mechanism with an enormous biological potential. It may, for example, represent a new way to modulate gene expression to the pathogen's advantage and to facilitate GI transfer. The microbes may have evolved a means to facilitate the “stealthy” transfer of genes encoding virulence factors that may be of benefit under new infection conditions

## Materials and Methods

### Bacterial strains, plasmids and culture media

Bacterial strains and plasmids used in this study are listed in Table 1. *Escherichia coli* strains were grown at 37°C in Luria Bertani (LB, Difco) media and *Pseudomonas* strains were grown at 25°C on Kings medium B (KB) [31], in LB broth or M9 minimal medium (M9,[32]). Antibiotics were used at the following concentrations (µg/ml): gentamicin (Gm) 10, kanamycin (Km) 50, ampicillin (Ap) 100, nitrofurantoin (NF) 100, and rifampicin (Rif) 100.

**Table 1:** Bacterial strains and plasmids.

Name	Genotype	Reference
***E. coli***		
39R861	Contains four plasmids used as molecular size standards – 154 kb, 66.2 kb, 37.6 kb and 7.2 kb	[37]
TOPO Top10	*E. coli* cloning strain	Invitrogen
***P. syringae*** ** pv. ** ***phaseolicola***		
1302A	Wild type strain containing PPHGI-1	[5]
SG120	1302A-PPHGI-1::eGFP, Km^R^	This study
SG126	1302A-PPHGI-1::eCFP, Km^R^	This study
SG248	1302A::Tn*7*-eGFP, Gm^R^	[22]
F341	SG126 plus chromosomal Tn7-eYFP, Gm^R^, Km^R^	This study
F532	SG122 plus chromosomal Tn7-dsRFP, Gm^R^, Km^R^	This study
**Plasmids**		
pRK2013	Helper plasmid for conjugation, Km^R^	[38]
AKN033	Vector containing Tn*7* containing eCFP, Ap^R^, Gm^R^	[39]
AKN069	Vector containing Tn*7* containing eYFP, Ap^R^, Gm^R^	[39]
AKN100	Vector containing Tn*7* containing eGFP, Ap^R^, Gm^R^	[39]
AKN132	Vector containing Tn*7* containing dsRFP, Ap^R^, Gm^R^	[39]
pUXBF13	Helper plasmid for Tn*7* transposition functions, Ap^R^	[40]
pK18mobsacB	suicide delivery vector containing *sacB*, Km^R^	[35]
ZERO-BLUNT-TOPO	Blunt end PCR cloning vector	Invitrogen
pSG028-1	ZERO-BLUNT-TOPO + OE-PCR-RCR (containing *Pac*I, *Pme*I, *Spe*I and *Asi*SI cloning sites) flanked by *Hin*dIII-*Xba*I	This study
pSG068-9	pK18mobsacB + *Hin*dIII-*Xba*I cloned NCR::OE-PCR-RCS, (containing *Pac*I, *Pme*I, *Spe*I and *Asi*SI cloning sites), Km^R^	This study
pSG097	*Pac*I-*Pme*I flanked *egfp* (ex AKN100) amplicon cloned in ZERO-BLUNT-TOPO, Km^R^	This study
pSG103	*Pac*I-*Pme*I flanked *ecfp* (ex AKN033) amplicon cloned in ZERO-BLUNT-TOPO, Km^R^	This study
pSG111	pSG068-9 containing *Pac*I-*Pme*I cloned eGFP, Km^R^	This study
pSG114	pSG068-9 containing *Pac*I-*Pme*I cloned eCFP, Km^R^	This study

Ap^R^, Rif^R^, Km^R^ indicate resistance to ampicillin, gentamicin, and kanamycin respectively.

### DNA isolation, PCR and plasmid extractions

Plasmid DNA was isolated from a pure bacterial culture using the QIAprep Spin Miniprep Kit (Qiagen) and restriction enzymatic digests performed as per manufacturers protocols (NEB biolabs). Unless stated otherwise, standard PCR reactions were performed using Phusion TM polymerase (NEB biolabs) using recommended cycling parameters with oligonucleotide primers (Table S1). Automated DNA sequencing was performed with an ABI 3130xl genetic analyser. *P. syringae* pv. *phaseolicola* plasmid profiles were determined by extracting total uncut plasmid DNA from overnight cultures as described previously [33].

### Insertion of eGFP/eCFP into PPHGI-1

Strand overlapping extension PCR [34] was used to introduce the rare cutting restriction enzyme sites *Pme*I and *Pac*I into a non-coding-region (NCR; 55667 bp – 56577 bp, Genbank accession AJ870974) of DNA within PPHGI-1 for introduction of eGFP or eCFP (Figure 1A). Three transcriptional terminators (TTs) in each translational frame were introduced upstream of *Pme*I and downstream of *Pac*I (Figure 1A). Overlapped PCR products were cloned into pK18mob*sacB* [35] to create pSG028-1. Both eGFP and eCFP were PCR amplified from AKN100 and AKN033 respectively using primers (F-pEXFP-*Pme*I and R-pEXP-*Pac*I, Table S1) that were designed to introduced *Pme*I and *Pac*I restriction sites respectively at amplicon terminal ends. PCR products were cloned into Zero-Blunt-Topo (Invitrogen) to create pSG097 (eGFP) and pSG103 (eCFP). *Pme*I and *Pac*I digests of pSG097 and pSG103 were respectively cloned into similarly digested pSG028-1 to create pSG111-eGFP and pSG114-eCFP. Introduction of the eGFP (pSG111-eGFP) and eCFP (pSG114-eCFP) into the NCR of PPHGI-1 was achieved by allelic exchange [36] with *P. syringae* pv. *phaseolicola* 1302A to create Km^R^ first cross-over construct strains, *P. syringae* pv. *phaseolicola* SG120 (eGFP) and *P. syringae* pv. *phaseolicola* SG126 (eCFP). *P. syringae* pv. *phaseolicola* SG120 and *P. syringae* pv. *phaseolicola* SG126 were confirmed to have *P. syringae* pv. *phaseolicola* 1302A wild type phenotypes as described previously [6].

### Chromosomal introduction of fluorescent proteins

dsRFP (AKN132, Table 1) and eYFP (AKN069) were introduced respectively into *P. syringae* pv. *phaseolicola* SG120 and *P. syringae* pv. *phaseolicola* SG126 via the Tn*7* transposon delivery system kindly provided by Lambertsen *et al*. [36] as described previously [22] to create *P. syringae* pv. *phaseolicola* F341 (*P. syringae* pv. *phaseolicola* 1302A with chromosomal eYFP and PPHGI-1::eCFP, Figure 1B) and *P. syringae* pv. *phaseolicola* F532 (*P. syringae* pv. *phaseolicola* 1302A with chromosomal dsRFP and PPHGI-1::eGFP, Figure 1C).

### *In planta* confocal imaging and data collection

*P. syringae* pv. *phaseolicola* inoculations and *in planta* confocal imaging of *P. syringae* pv. *phaseolicola* fluorescence in *P. vulgaris* was performed essentially as described previously [22]. Confocal visualisation of *in planta* infiltration of dual-labelled strains (F532 and F341) was performed on the Leica TCS-SP2-DM IRE2 confocal laser scanning microscope (Leica Microsystems Wetzlar GmbH) at 25× or 40× (objective magnification) for colony morphology and 63× or 100× for visualisation of individual cell dispersal. Variable AOTF filters were used for the following fluorophores (excitation/emission): eYFP (514 nm/525–600 nm); eCFP (440 nm/465–495 nm); eGFP (488 nm/516–539 nm); dsRFP (568 nm/600–644 nm); and for plant autofluorescence (440 nm, 650–785 nm). Z-series imaging was performed at intervals of 0.3 µm (individual cells) and 1 µm (colonies). All confocal images were assigned false colour in images (eCFP cyan, eGFP green, eYFP yellow, dsRFP red and plant tissue blue). 1 µm z-section scan intervals were used to ensure all bacterial fluorescence was analysed. Three 5 mm^2^ leaf samples were analysed from each variable and each sample had at least 12 random areas confocal imaged to ensure representative data.

### *P. syringae* pv. *phaseolicola* growth and visualisation on agarose slides

Glass slides were layered with approximately 0.17 mm of agarose (2.2% final volume) containing desired liquid (ddH_2_O, M9, or apoplastic fluid) and used immediately. Apoplastic fluid was extracted from 10 day old TG or CW bean leaves, infiltrated CW leaves undergoing disease or TG leaves undergoing the HR as described previously [17]. All apoplastic fluids were used immediately after preparation following filter-sterilization (0.2 µm). Overnight cultures were washed and serially diluted in ¼ Ringers solution to OD_600_ equivalent of: 1×10^−2^, 10^−4^, 10^−6^, or 10^−8^. Inoculum droplets (10 µl) were placed onto respective agarose slides and incubated within a sterile sealed container (containing high humidity) at 25°C for 24 h–144 h as required. The optimal micro-colony development was usually observed at 1×10^−4^ OD_600_ after 24 h on M9/TG (1:1) apoplastic fluid. Cover slips were added immediately prior to confocal imaging using the Zeiss Axiovert 200 in conjunction with the Ultraview FRET H rapid confocal imaging system (Perkin Elmer Instruments Ltd) as described previously [22].

### Agarose recovery of *P. syringae* pv. *phaseolicola* F532 cells with loss of PPHGI-1::eGFP fluorescence

Slide preparations of *P. syringae* pv. *phaseolicola* F532 were inoculated (1×10^−4^ OD_600_) onto M9/TG slides for 48 h. Agarose slides were imaged using both dsRFP and eGFP channels to ensure *P. syringae* pv. *phaseolicola* F532 cells had loss of PPHGI-1::eGFP fluorescence (such as Fig. 3Av). Agarose from the imaged slide was immediately re-suspended into ¼ Ringers by Eppendorf-pestle homogenization and vortex mixing. Extracts were serially diluted onto KB agar and incubated (25°C, 48 h). Resulting Gm^R^ colonies were replica plated onto KB vs. KB+Gm (to select for Tn*7*-dsRFP) and KB+Gm+Km (to select for Tn*7*-dsRFP and PPHGI-1::eGFP). After 48 h at 25°C, all colonies were visualised for eGFP fluorescence and subjected to TG pod-stab assay (to determine the presence of *avrPphB*, and therefore PPHGI-1) by analysis of HR/disease phenotype as described previously [6]

### Fluorescence-activated cell sorting (FACS) separation of *P. syringae* pv. *phaseolicola* F532 populations based on eGFP fluorescence

*P. syringae* pv. *phaseolicola* F532 was grown in broth cultures consisting of: M9/TG apoplastic fluid (1:1). 200,000 particles (events) were collected for both F532/GFP+ and F532/GFP- and samples were serially diluted and plated onto KB+Gm to correlate FACS events with viable *P. syringae* pv. *phaseolicola* cell recovery. FACS was performed on either (1) a FACSVantage cell sorter (Becton Dickinson), equipped with a 488 nm argon laser; or (2) an Influx cell sorter (Becton Dickinson). *P. syringae* pv. *phaseolicola* F532 cultures were separated based on GFP fluorescence (F532/GFP+) and non-GFP fluorescence (F532/GFP-). Both F532/GFP+ and F532/GFP- FACS sub-populations were analysed immediately using confocal microscopy to confirm status of eGFP and dsRFP fluorescence.

### qPCR of *xerC* and *avrPphB* expression and circular episome (*CE*) production

qPCR was used to quantify *xerC* and *avrPphB* expression and PPHGI-1 *CE* production *in vitro* and *in planta*. *P. syringae* pv. *phaseolicola* strains were added to respective growth medium (either LB, M9, apoplastic fluid and/or M9/apoplastic fluid mixes) and after 5 h, cells were harvested and gene expression stopped using RNA protect reagent (Qiagen, UK) or DNA lysis solution (Gentra Systems, UK). For quantification of *xerC*, *avrPphB* and *gyrB* expression, RNA was extracted using the RNAeasy kit (Qiagen, UK) followed by a second DNase step of 15 min at 37°C (Promega, UK). cDNA was synthesised using the TaqMan Reverse Transcription Reagents kit (Applied Biosystems). For quantification of the PPHGI-1 *CE*, DNA was extracted using the Puregene DNA isolation kit (Gentra Systems, UK). Triplicate samples were taken for each variable for cDNA/DNA synthesis, and each sample was analyzed twice in separate amplifications. cDNA and DNA were quantified using a Nanodrop spectrophotometer (Thermo Scientific, USA) and adjusted to 100 ng/µl.

When gene expression was analysed after FACS separation, cells were sorted directly into either RNA protect reagent (Qiagen UK) or DNA lysis solution (Gentra Systems, UK) and RNA/DNA extracted as above. qPCR was performed on an ABI 7300 Real-Time PCR System (Applied Biosystems), calibrated using 7300 Real-Time PCR Systems Spectral Calibration kit (Applied Biosystems) and probes (Table S1) were labelled with 3′ FAM and 5′ TAMRA TaqMan dyes. Reaction volume (25 µl) consisted of 12.5 µl TaqMan PCR mastermix (Applied Biosystems), 2 µl each primer (10 µM), 2 µl probe (5 µM) and 6.5 µl RNase free water. Standard qPCR cycling conditions were 50°C for 2 min, 95°C for 10 min and 40× cycles of 95°C for 15 sec followed by 60°C for 1 min. Results were analysed using ABI 7300 System SDS software (Applied Biosystems) and compared using JMP IN 7.0 statistical analysis software (www.jmpin.com). Average abundance of *xerC* and *avrPphB* RNA and *CE* DNA were expressed relative to levels of *gyrB*.

## Supporting Information

Table S1 Oligonucleotide primers used in this study.
(DOC)
