# Supplementary material for: The Stealth Episome: Suppression of Gene Expression on the Excised Genomic Island PPHGI-1 from Pseudomonas syringae pv. phaseolicola
Source: PLoS Pathog. 2011 Mar 31;7(3):e1002010. doi: 10.1371/journal.ppat.1002010 (PMC3068993; doi:10.1371/journal.ppat.1002010)
Supplement: Table S1 — Oligonucleotide primers used in this study. (DOC) [file ppat.1002010.s001.doc]

**Table S1. Oligonucleotide primers used in this study**

| **Primer Name** | **Primer Sequence 5'–3'** | **Gene or Location on *PPH*GI-1** | **Reference** |
| --- | --- | --- | --- |
|  |  |  |  |
| ***PCR*** |  |  |  |
| pK18-Seq-P1 | GCCAGCTGGCGAAAGGGGGATGTGC | Sequencing primer to check pK18mob*sacB* inserts | This study |
| pK18-Seq-P2 | GCTTTACACTTTATGCTTCCGGCTCG | Sequencing primer to check pK18mob*sacB* inserts | This study |
| NCR-F-*Hind*III | CCCaagcttGAGTCCAACGTACCTTGCTCG | NCR OE-PCR complement primer. Addition of *Hind*III | This study |
| NCR-R-*Xba*I | TGCtctagaCGAACACATCTTCTGTAGCTGCG | NCR OE-PCR complement primer. Addition of *Xba*I | This study |
| NCR-RCS R-OE01 | ACTAGTCTAGTTAATTACTTAATTAACGTACGTTTAAACCTAGTTAATTAGAAACAAAGGGGTGGTTTCA | NCR OE PCR primer. NCR overlap includes *PmeI*, *PacI*, *SpeI*, *Asi*SI | This study |
| NCR-RCS F-OE02 | TAATTAAGTAATTAACTAGACTAGTACGTAGCGATCGCTAATTAACTAGCATTGATGTGGATGCTTGGA | NCR OE PCR primer. NCR overlap includes *Pme*I, *Pac*I, *Spe*I, *Asi*SI | This study |
| NCR_RCS_F_CK_01 | TGAAACCACCCCTTTGTTTC | Sequencing primers to check inserts into NCR of sg068-9 | This study |
| NCR_RCS_R_CK_02 | TCCAAGCATCCACATCAATG | Sequencing primers to check inserts into NCR of sg068-9 | This study |
| F-pEXFP-*Pme*I | CGGTTTAAACATTTATCAGGGTTATTGTCT | Amplification of fluorescent proteins (either eGFP, eCFP, eYFP or dsRFP) from Tn*7* vectors. Addition of *Pme*I | This study |
| R-pEXP-*Pac*I | GGTTAATTAAATTACTTGTACAGCTCGTCCATGCC | Amplification of fluorescent proteins (either eGFP, eCFP, eYFP or dsRFP) from Tn*7* vectors. addition of *Pac*I | This study |
| LJatt | ATGTGCGATGAGGTCGAATATGC | Circular intermediate | [1] |
| RJatt | TATGCGTGGCCTCCAGTAGCTCTG | Circular intermediate | [1] |
|  |  |  |  |
| ***qPCR*** |  |  |  |
| QCI-F | CATGGGCCTTCCAGATTTTC | qPCR circular intermediate primer | This study |
| QCI-R | CTGCGGTTTGGGATACTGAAC | qPCR circular intermediate primer | This study |
| QCI-P | *CGTAACGCTGAGGCAGGCCCC* | qPCR circular intermediate probe | This study |
| *gyrB*-F | GATGATGGAATCGGTGTCGAA | qPCR gyrase B primer | This study |
| *gyrB*-R | TTGGTGAAGCACAACAGGTTCT | qPCR gyrase B primer | This study |
| *gyrB*-P | *CCCTGCAGTGGAACGACAGCTTCA* | qPCR gyrase B probe | This study |
| *xerC*Q-F | CGACGATACGGCCTCCAA | qPCR *xerC* primer | [1] |
| *xerC*Q-R | AAAGGTGCGGTCGACATCA | qPCR *xerC* primer | [1] |
| *xerC*Q-P | *CCCCCTATAGCGGAGCGTCTGGAA* | qPCR *xerC* probe | [1] |
| avrPphBQ-F | CCCATTCCTGGCAATGACA | qPCR *avrPphB* primer | This study |
| avrPphBQ-R | TTACGCCTGAAGAGGATGCA | qPCR *avrPphB* primer | This study |
| avrPphBQ-P | *TGGGCGATAAAGGG* | qPCR *avrPphB* probe | This study |

* Probes labelled with 5' FAM and 3' TAMRA TaqMan dyes***)***

**1.** Pitman AR, Jackson RW, Mansfield JW, Kaitell V, Thwaites R, Arnold DL (2005) Exposure to host resistance mechanisms drives evolution of bacterial virulence in plants. Current Biology 15: 2230-2235.
